# Supplementary material for: DNA methylation governs the sensitivity of repeats to restriction by the HUSH-MORC2 corepressor
Source: Nat Commun. 2024 Aug 30;15:7534. doi: 10.1038/s41467-024-50765-4 (PMC11364546; doi:10.1038/s41467-024-50765-4)
Supplement: Supplementary file 1 — Supplementary Information [file 41467_2024_50765_MOESM1_ESM.pdf]

Supplementary information:

'DNA methylation governs the sensitivity of repeats to restriction by the HUSH-MORC2 corepressor' by Pandiloski et al.

SUPPLEMENTARY FIGURES

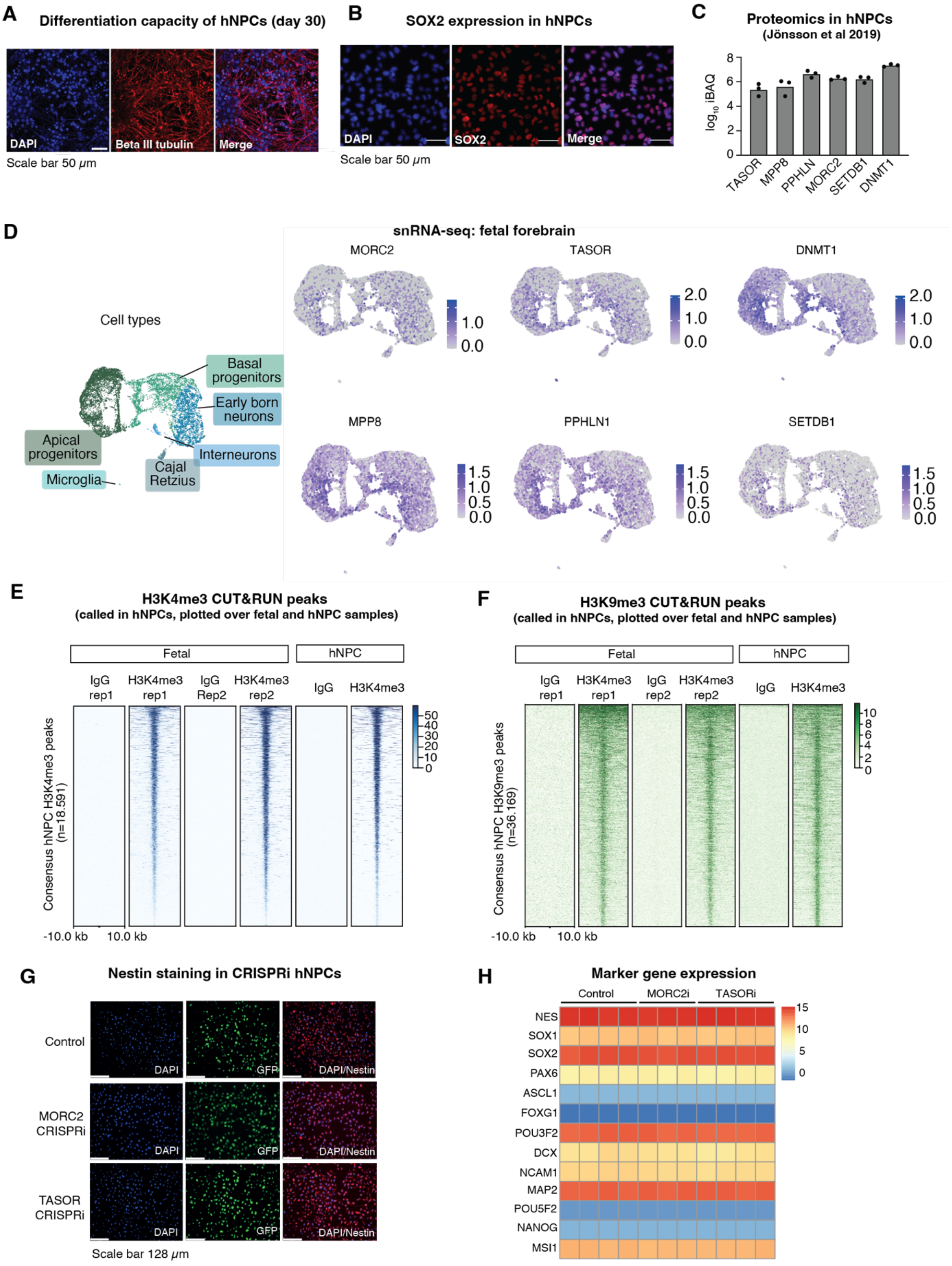

Supplementary Figure 1. (A) Immunostaining of neurons differentiated from the hNPC line used in this study illustrating expression of neuronal marker beta-III-tubulin, imaged at day 30. **(B)** Immunostaining of the hNPC line used in this study illustrating SOX2 expression. **(C)** Detection of epigenetic repeat regulators in published hNPC proteomic analysis.[37] **(D)** Expression of HUSH complex

components, MORC2, SETDB1 and DNMT1 in the developing human forebrain according to snRNA-seq analysis (n=6, 7-10 weeks post-conception, data from GSE224747).[8] **(E, F)** Heatmaps illustrating CUT&RUN signal enrichment of H3K4me3, H3K9me3 and a non-targeting IgG control in fetal forebrain samples (n=2) and hNPCs plotted over consensus peaks called in hNPC samples. Displayed are the genomic regions spanning +/- 10kb from the peak centre. Experiments on hNPC samples were repeated at least three times with similar results. **(G)** Representative immunostaining to assess dCas9-KRAB-T2A-GFP and Nestin expression in MORC2 and TASOR CRISPRi and control hNPCs. **(H)** Heatmap of normalized, log2-transformed counts of marker gene expression based on RNA-seq data from control hNPCs (n=4), MORC2 CRISPRi hNPCs (n=3) and TASOR CRISPRi hNPCs (n=4), illustrating that cell identity was not measurably perturbed at a transcriptomic level by the depletion of HUSH-MORC2. Source data are provided in a Source Data File.

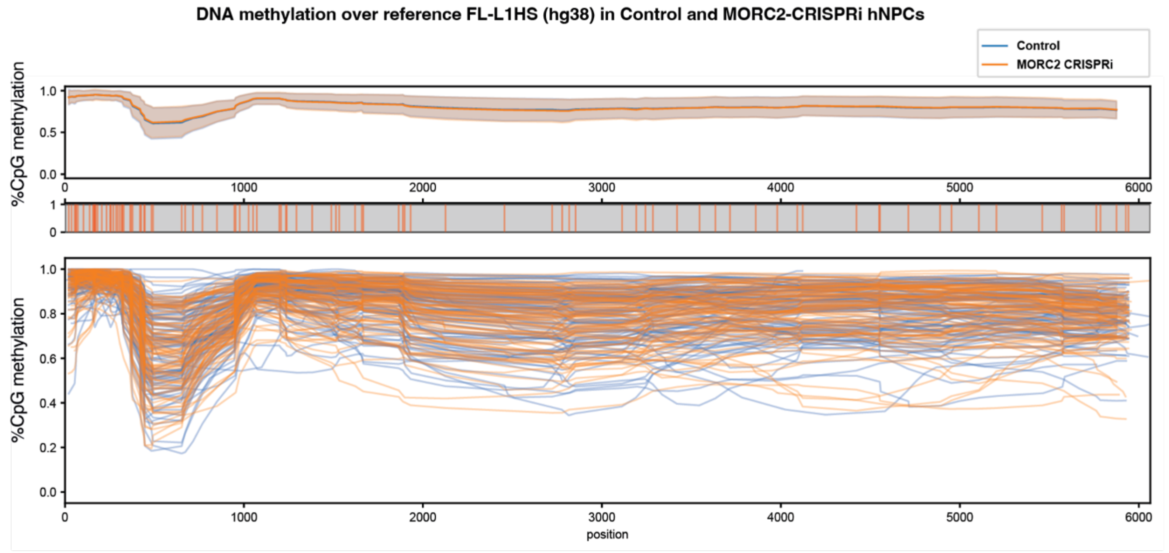

Supplementary Figure 2. Summary plot of methylation status of all reference full-length (>6kb) L1HS in the hg38 reference genome (n=302) based on Nanopore sequencing data in Control (blue lines) and MORC2 CRISPRi (orange) hNPCs. Whole genome sequencing was performed once for each condition.

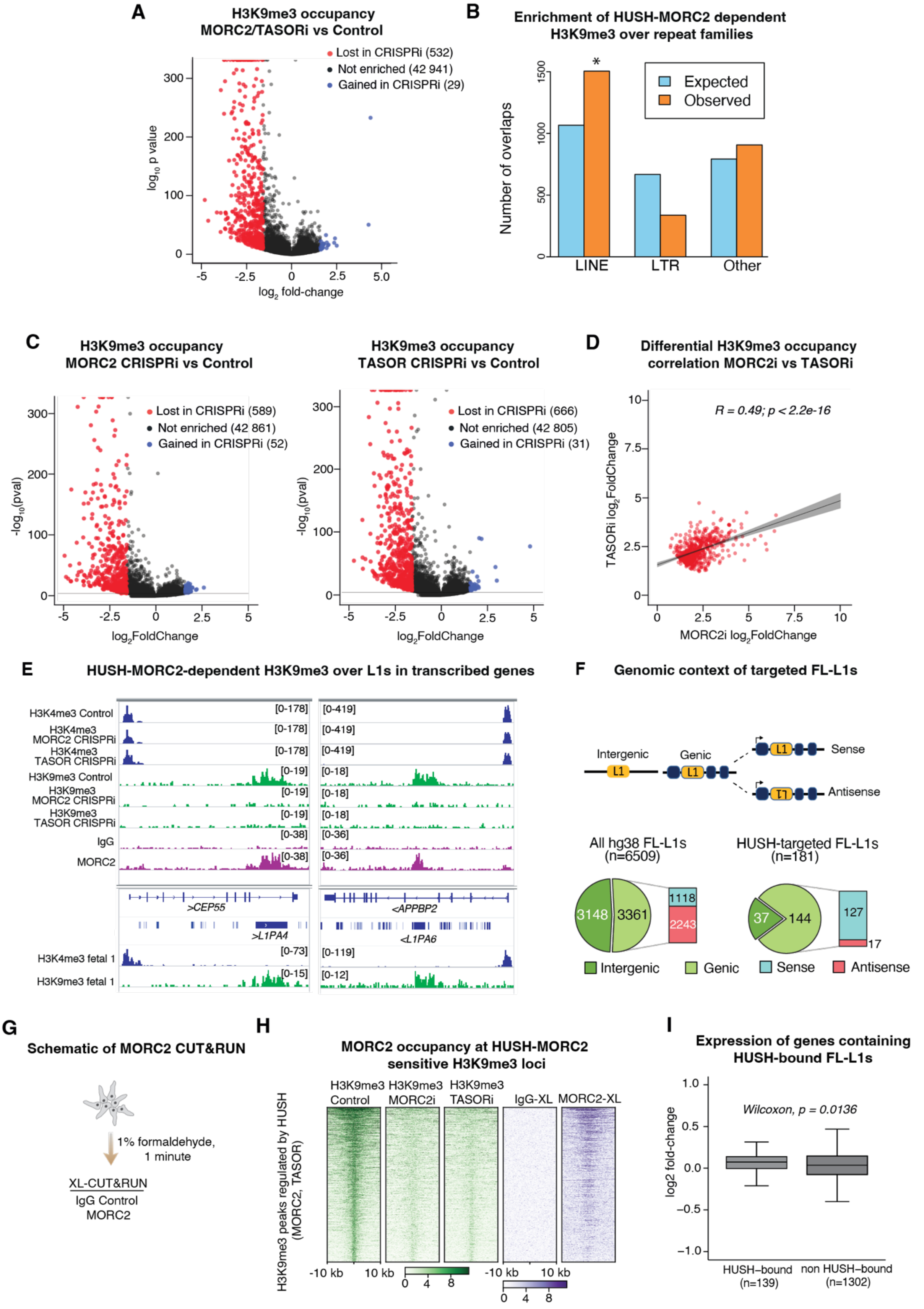

Supplementary Figure 3. (A) Volcano plot showing H3K9me3 changes genome-wide over H3K9me3 peaks in MORC2 (n=2) and TASOR (n=2) CRISPRi hNPCs compared to controls (n=4) as measured by CUT&RUN epigenome profiling 10 days post-transduction (significant points defined by fold-

change>3 and Poisson p-value <1e-04; MORC2 and TASOR CRISPRi treatments grouped in the differential analysis). **(B)** Top repeat class overlaps with regions losing H3K9me3 upon MORC2 and TASOR depletion. * p=1.04E-9, chi-squared test. **(C)** The same analysis as in panel A, but with MORC2 and TASOR CRISPRi data kept as separate treatment groups in the differential analysis. **(D)** Correlation analysis between H3K9me3 changes in TASOR and MORC2 CRISPRi treatments relative to controls. **(E)** Genome browser snapshot examples of intronic L1PA elements occupying the same strand as a transcribed host gene, bound by MORC2 and losing H3K9me3 upon MORC2 and TASOR depletion. **(F)** Summary of genomic attributes of full-length (FL) L1s losing H3K9me3 upon MORC2 and TASOR depletion illustrating a preference of genic, sense L1 elements. Also shown is the genome-wide distribution. **(G)** Schematic of MORC2 CUT&RUN experiments in hNPCs using a crosslinking protocol. **(H)** Presence of MORC2 over TASOR and MORC2-controlled H3K9me3 peaks in hNPCs. Displayed are the genomic regions spanning +/- 10kb from the peak centre. **(I)** Comparison of genes expression changes upon MORC2 and TASOR CRISPRi between genes containing FL-L1s marked by TASOR and MORC2-dependent H3K9me3 and genes containing non-targeted FL-L1s. The central bands denote medians. Boxes represent the interquartile range (IQR). Whiskers extend 1.5x IQR beyond the box. Wilcoxon rank sum and signed rank test was used.

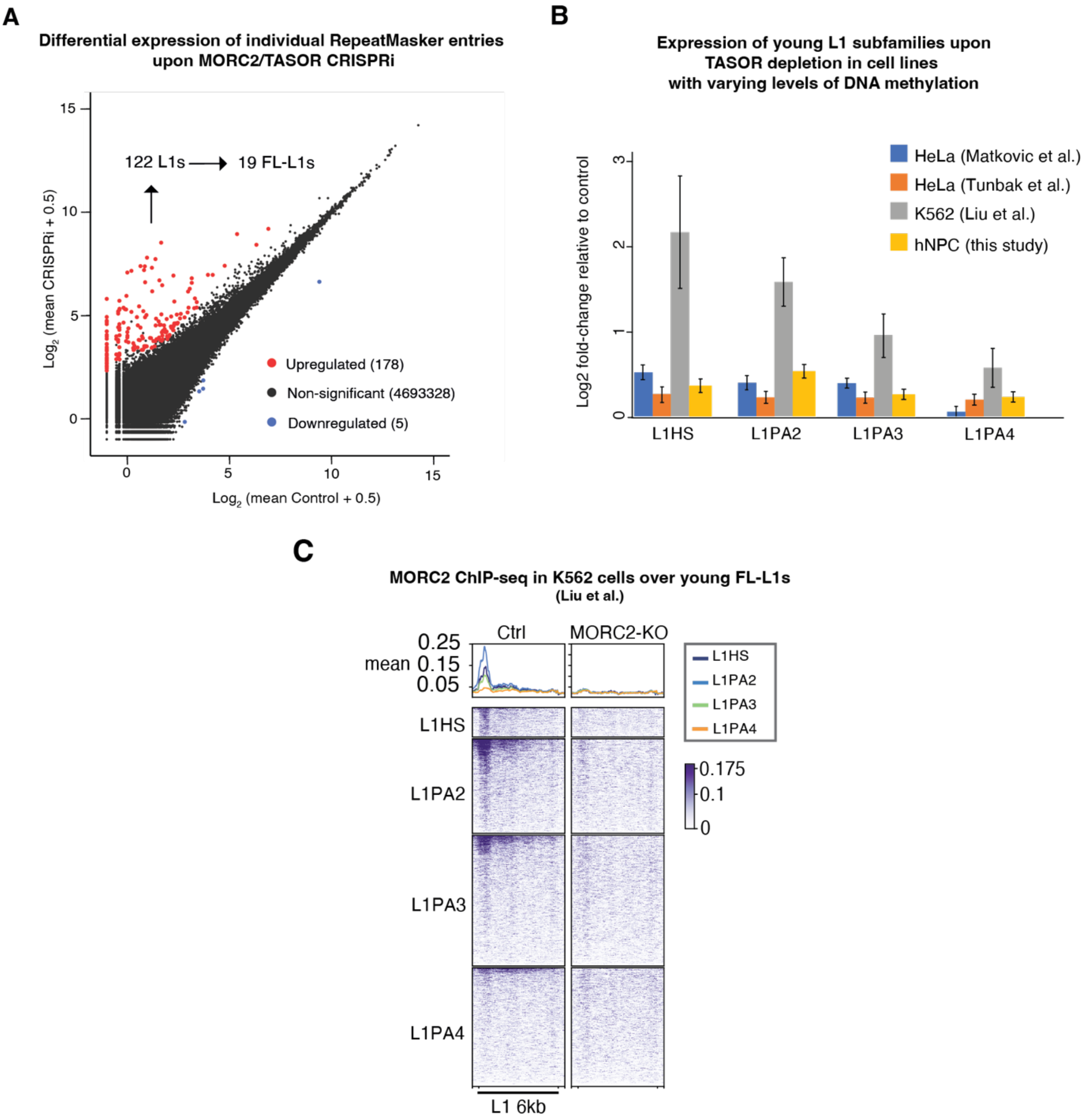

Supplementary Figure 4. (A) Mean plot illustrating differential expression analysis of individual repeats from RepeatMasker in MORC2 and TASOR CRISPRi treatments (n=7) versus control hNPCs (n=4). Elements with |log2 fold-change| >2 and padj <0.05 are highlighted. The number of upregulated L1s and FL-L1s are given. **(B)** Effect of TASOR depletion on transcription of young L1 subfamilies in different cell lines (quantified by TEtranscripts, presented as log2-fold change (LFC) +/- standard error). Data are from Tunbak/Rowe et al. 2020, Matkovix/Margottin-Goguet et al. 2022, Liu/Wysocka et al. 2018 (GSE135765; GSE184399; GSE95374). **(C)** MORC2 binding to evolutionarily young FL-L1s in the demethylated K562 line, assessed by ChIP-seq in Ctrl and MORC2-KO cells. Data from Liu/Wysocka et al. 2018 (GSE95374).

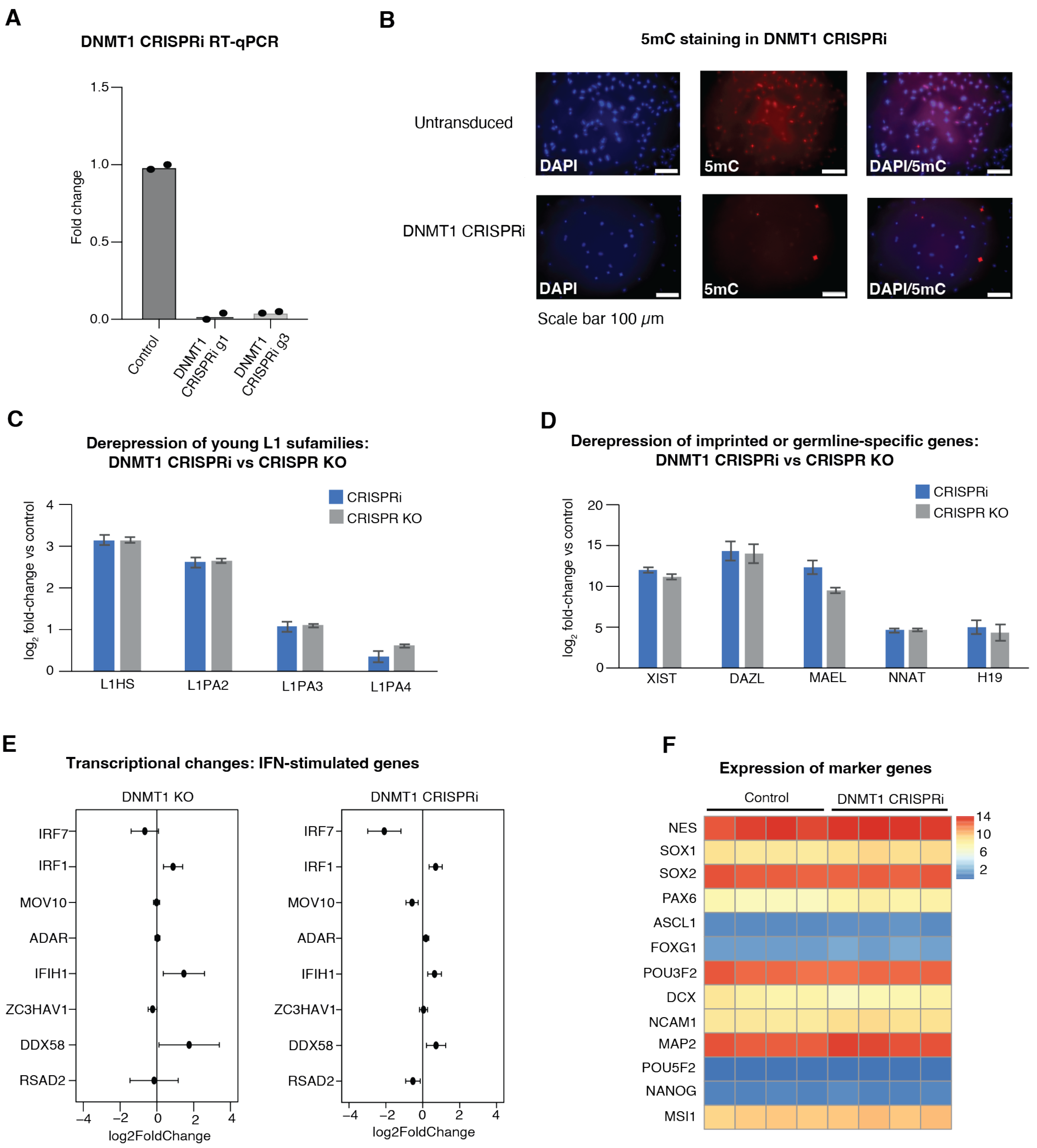

Supplementary Figure 5. (A) Reverse transcription quantitative PCR (RT-qPCR) of DNMT1 transcript levels in DNMT1 CRISPRi cells, relative to a non-targeting control, 10 days post-transduction. Shown are data from two independent experiments, with points representing the mean of three technical replicates in each case. **(B)** Representative 5mC immunostaining in untransduced and DNMT1 CRISPRi cells illustrating global loss of DNA methylation. Experiment was repeated once with similar results. **(C)** TEtranscripts log_2-fold-change (LFC) of young L1 subfamilies in DNMT1 CRISPRi (n=4) versus control hNPCs (n=4) and DNMT1-KO (n=3) versus control hNPCs (n=3). DNMT1-KO data taken from Jönsson et al. (2019). In each case the error bars represent +/- LFC standard error calculated by DESeq2 taking all samples into account. **(D)** LFC of imprinted and germline-specific genes in DNMT1 CRISPRi (n=4) versus control hNPCs (n=4) and DNMT1-KO (n=3) versus control hNPCs (n=3). DNMT1-KO data taken from Jönsson et al. (2019). In each case the error bars represent +/- LFC standard error calculated by DESeq2 taking all samples into account. **(E)** Differential expression analysis of selected interferon (IFN) stimulated genes in then in DNMT1-KO hNPCs (n=3) versus control (n=3) hNPCs and DNMT1 CRISPRi treatments (n=4) versus control hNPCs (n=4). The DNMT1-KO RNA-seq data was taken from Jönsson et al. (2019). Shown are LFC values +/- LFC standard error

calculated by DESeq2, taking all samples into account. (**F**) Heatmap of normalized, log2-transformed counts of marker gene expression based on RNA-seq data from control hNPCs (n=4) and DNMT1 CRISPRi hNPCs (n=4). Source data are provided in a Source Data file.

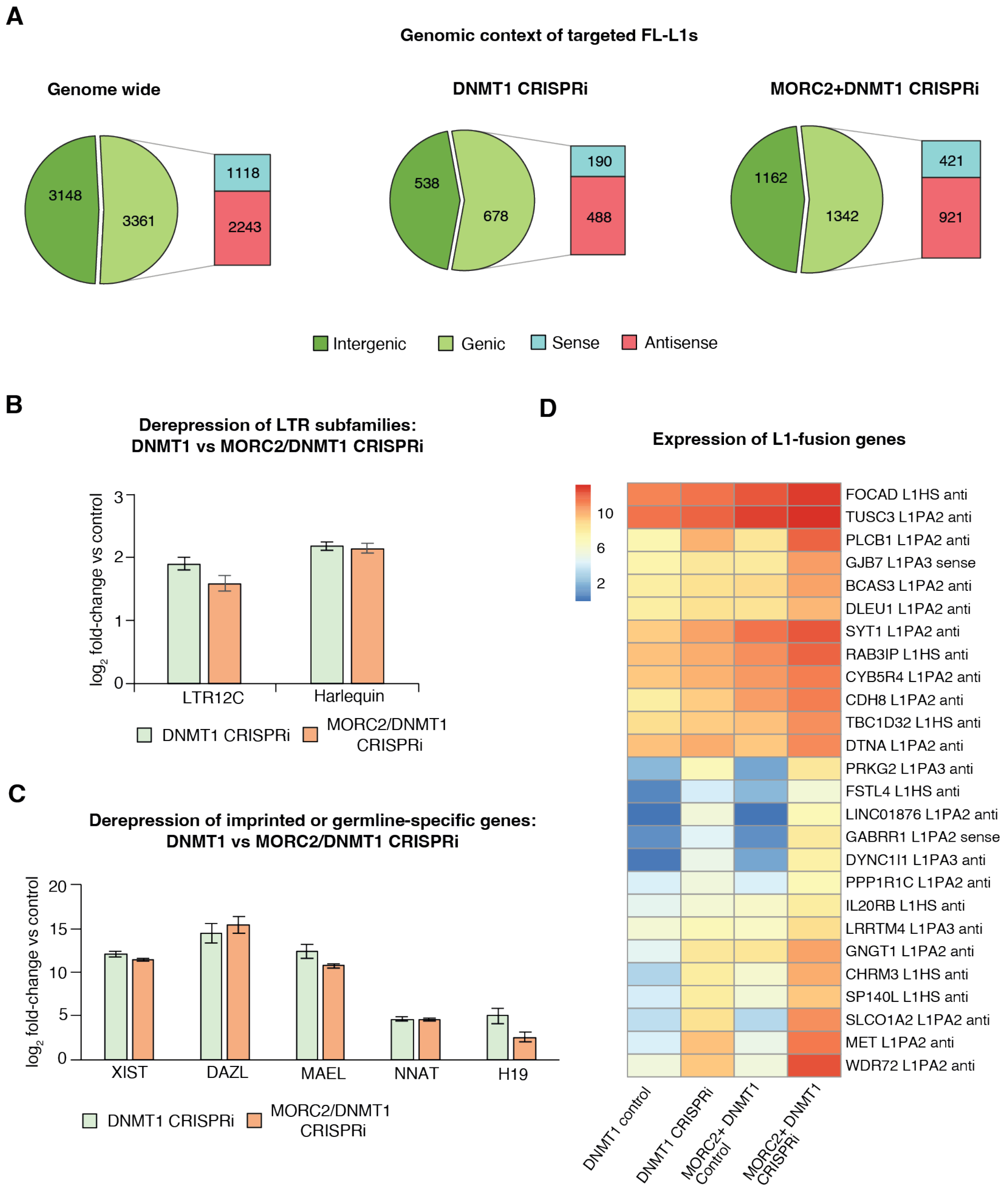

Supplementary Figure 6. (A) Summary of genomic attributes of full-length (FL, defined as >6kb) L1s activated upon DNMT1 and MORC2/DNMT1 CRISPRi depletion. Genic refers to at least 1 bp overlap with an annotated gene, otherwise elements were classified as intergenic. Amongst genic elements, these were classified as 'sense' or 'antisense' with respect to the host gene if they occupied the same or opposite DNA strand, respectively. **(B)** TEtranscripts log_2-fold-change (LFC) of selected DNMT1-controlled LTR families measured by RNA-seq in DNMT1 CRISPRi (n=4, green) and MORC2/DNMT1 double CRISPRi (n=4, orange) versus controls (n=4 for each experiment). **(C)** LFC of imprinted and germline-specific genes in DNMT1 CRISPRi (n=4, green) and MORC2/DNMT1 double CRISPRi (n=4, orange) versus controls (n=4 for each experiment). In each case the error bars represent +/- LFC standard error calculated by DESeq2 taking all samples into account. **(D)** Heatmap of normalized read counts of L1-fusion genes measured by RNA-seq in DNMT1 CRISPRi (n=4) and MORC2/DNMT1 double CRISPRi (n=4) versus controls (n=4 for each experiment). Given next to each row are the subfamily to which the L1 belongs and whether it occupies the same (sense) or opposite (anti) strand relative to the gene. The list of fusion genes comes from Jönsson et al (2019).

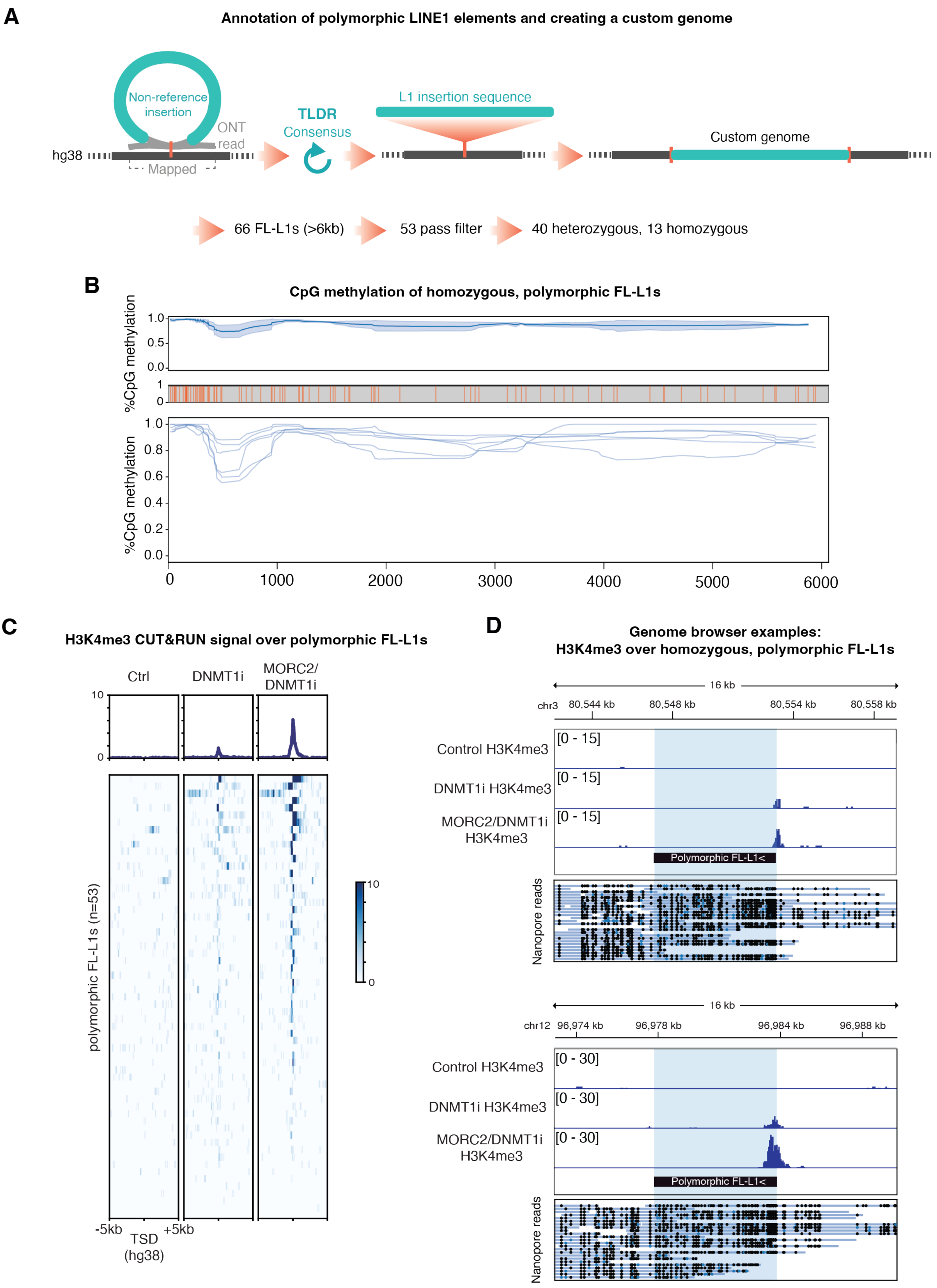

Supplementary Figure 7. (A) Schematic illustration of calling non-reference L1s in the hNPC genome based on Oxford Nanopore sequencing data. Control and MORC2 CRISPRi hNPCs were both used and only elements called in both datasets were considered real. **(B)** Summary plot of methylation status of polymorphic homozygous full-length (>6kb) L1HS (n=13). **(C)** CUT&RUN heatmap of H3K4me3 signal from Control, DNMT1-CRISPRi and MORC2/DNMT1-CRISPRi hNPCs, centred on the hg38-

reference target-site duplication (TSD) coordinates identified by TLDR, leveraging the signal that spreads to the surrounding reference genome up- or down-stream of the TSD in polymorphic FL-L1s (n=53, both heterozygous and homozygous alleles are included). **(D)** Two genome browser snapshots of H3K4me3 and Nanopore DNA methylation data mapped onto the custom-built genome at polymorphic homozygous FL-L1s, illustrating epigenetic regulation of these individual integrants. Only uniquely-mapping reads were retained. CpGs called as methylated in Nanopore reads are depicted as filled black circles; unmethylated CpGs as open blue circles. CUT&RUN experiments were repeated once with similar results.

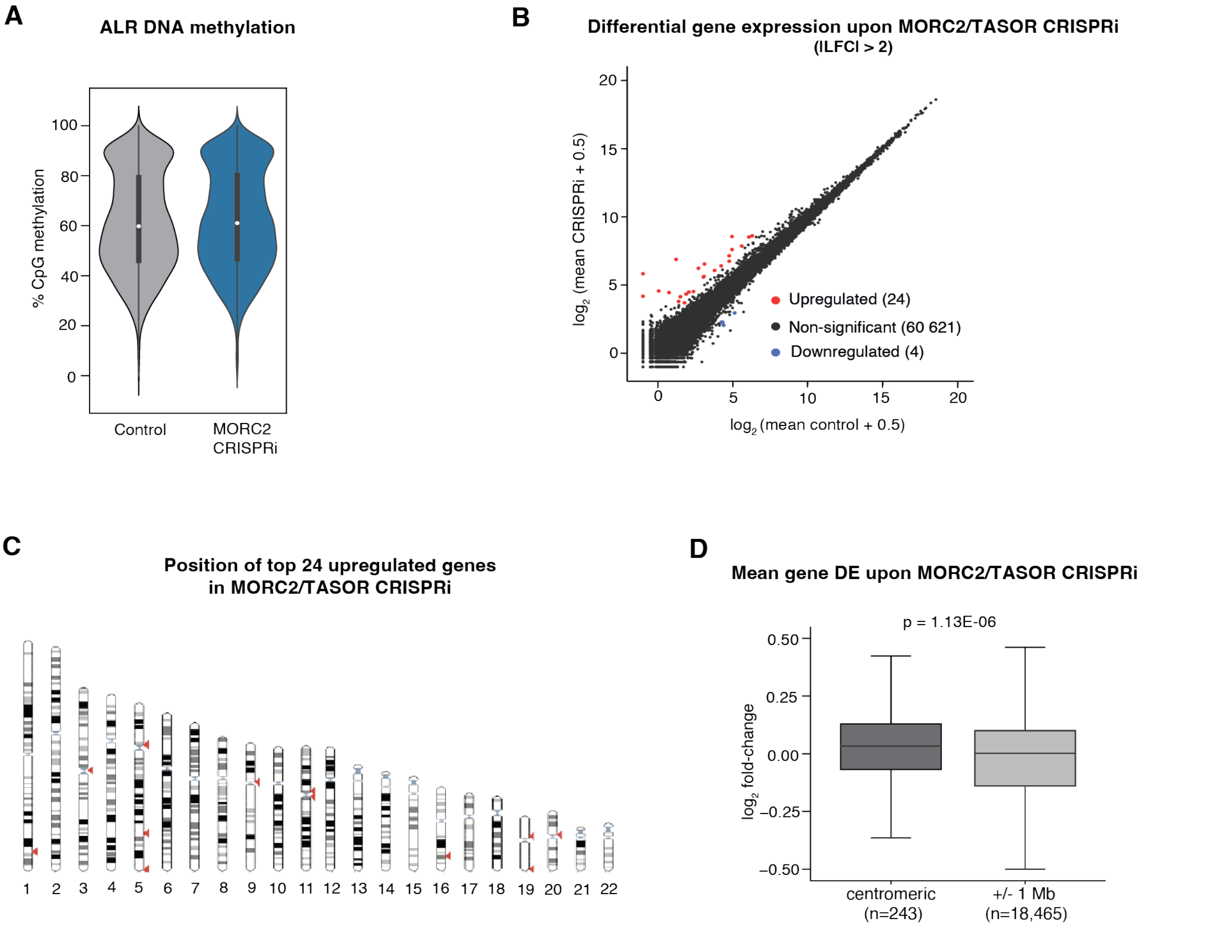

Supplementary Figure 8. (A) Average CpG methylation in reads mapping uniquely to pericentromeric regions in whole-genome Nanopore sequencing data from control (n=1) and MORC2 CRISPRi hNPCs (n=1). **(B)** Differential gene expression in MORC2 and TASOR CRISPRi (n=7) versus control hNPCs (n=4). Elements with $|\log_2$ fold-change$|$ >2 and padj <0.05 are highlighted. **(C)** Chromosome positions of top upregulated autosomal genes in MORC2 and TASOR CRISPRi hNPCs, illustrating a clustering of genes near centromeres. **(D)** Comparison of gene expression changes upon MORC2 and TASOR CRISPRi between genes within 1 Mb of an autosomal centromere and those outside. Central bands denote medians. Boxes represent the interquartile range (IQR). Whiskers extend 1.5x IQR beyond the box. Statistical test: Wilcoxon rank sum with signed rank. Source Data are provided in a Source Data file.

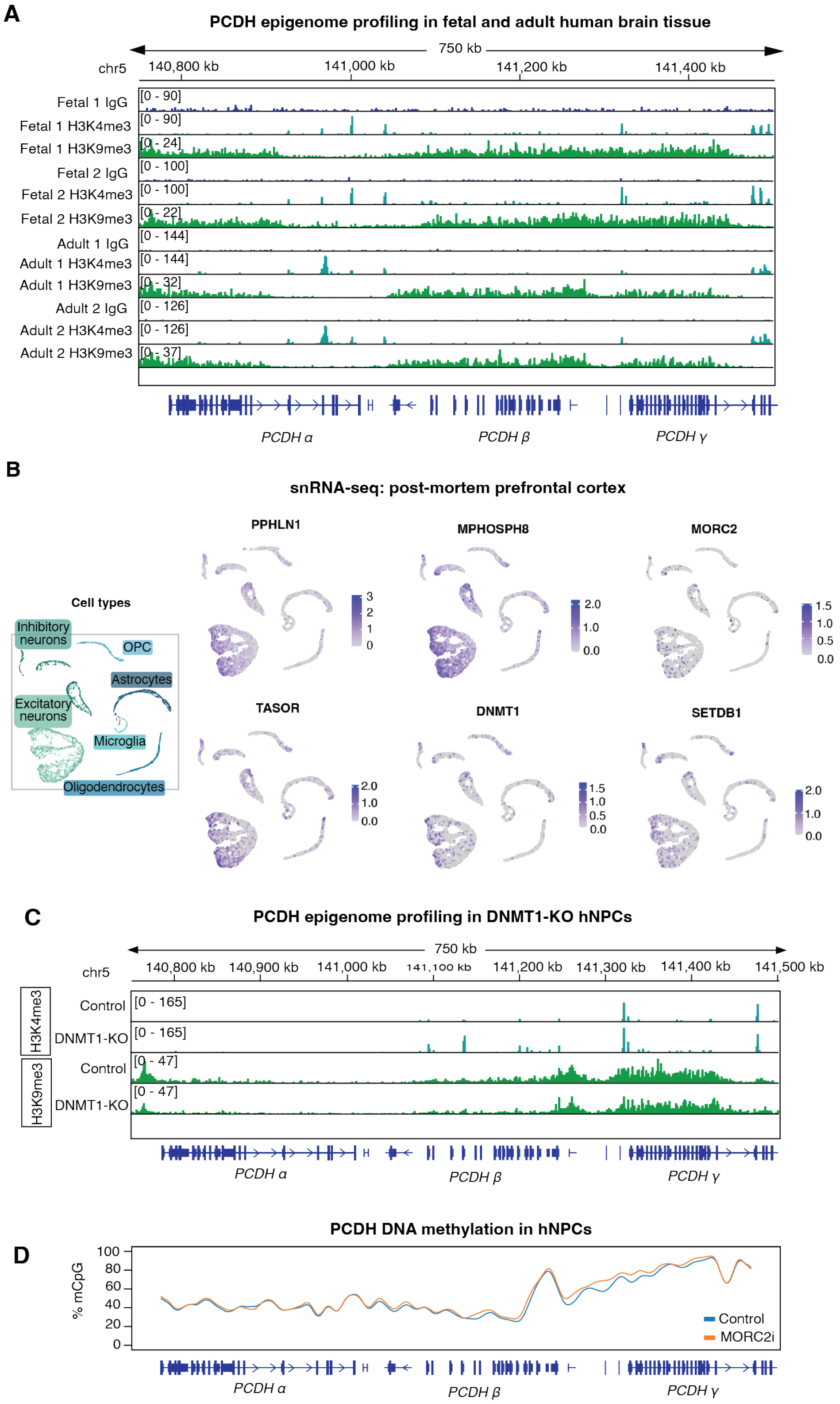

Supplementary Figure 9. (A) Genome browser snapshot illustrating epigenomic profiling of clustered protocadherins (*PCDH*) in fetal (n=2) and adult post-mortem (n=2) human brain tissue. For adult tissue, NeuN+ nuclei were sorted prior to CUT&RUN (see Methods). **(B)** Expression of epigenetic repeat regulators in snRNA-seq analysis of post-mortem human cortical tissue (n=5). Data from GSE209552. **(C)** H3K4me3 and H3K9me3 profiles in control and DNMT1-KO hNPCs over clustered *PCDH* genes. Experiments were repeated twice with similar results. **(D)** DNA methylation profile of control (n=1) and MORC2 CRISPRi hNPCs (n=1) according to whole-genome Oxford Nanopore Sequencing analysis.

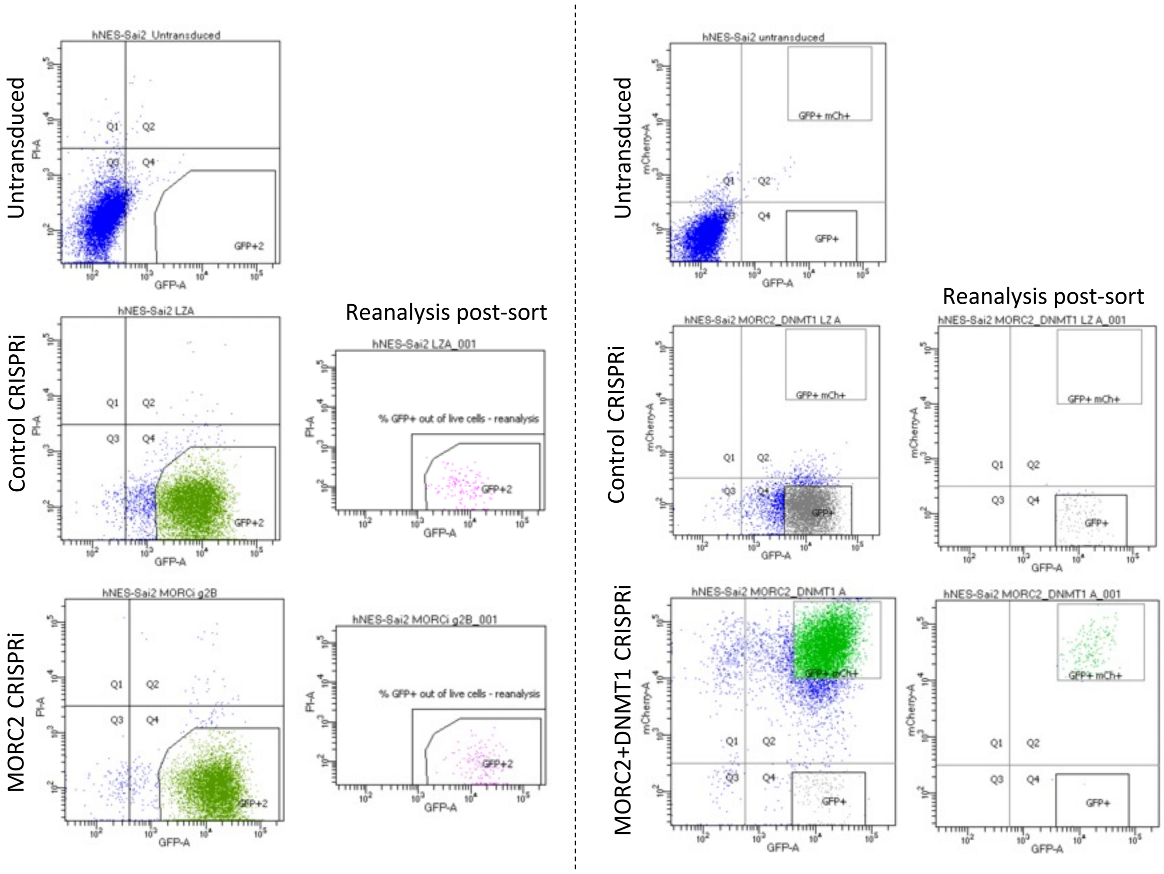

Supplementary Figure10. FACS gating strategies for isolating GFP-positive (left) or GFP/mCherry double positive (right) hNPCs in this study. Shown as insets are purity assessments made by reanalysing a subset of sorted cells.
