# Supplementary material for: DNA methylation governs the sensitivity of repeats to restriction by the HUSH-MORC2 corepressor
Source: Nat Commun. 2024 Aug 30;15:7534. doi: 10.1038/s41467-024-50765-4 (PMC11364546; doi:10.1038/s41467-024-50765-4)
Supplement: Supplementary file 3 — Reporting Summary [file 41467_2024_50765_MOESM3_ESM.pdf]

nature portfolio

Corresponding author(s): Christopher Douse
Last updated by author(s): July 8th 2024

Reporting Summary

Nature Portfolio wishes to improve the reproducibility of the work that we publish. This form provides structure for consistency and transparency in reporting. For further information on Nature Portfolio policies, see our Editorial Policies and the Editorial Policy Checklist.

Statistics

For all statistical analyses, confirm that the following items are present in the figure legend, table legend, main text, or Methods section.

n/a	Confirmed	
☐	☒	The exact sample size (*n*) for each experimental group/condition, given as a discrete number and unit of measurement
☐	☒	A statement on whether measurements were taken from distinct samples or whether the same sample was measured repeatedly
☐	☒	The statistical test(s) used AND whether they are one- or two-sided<br>*Only common tests should be described solely by name; describe more complex techniques in the Methods section.*
☒	☐	A description of all covariates tested
☒	☐	A description of any assumptions or corrections, such as tests of normality and adjustment for multiple comparisons
☐	☒	A full description of the statistical parameters including central tendency (e.g. means) or other basic estimates (e.g. regression coefficient) AND variation (e.g. standard deviation) or associated estimates of uncertainty (e.g. confidence intervals)
☐	☒	For null hypothesis testing, the test statistic (e.g. *F*, *t*, *r*) with confidence intervals, effect sizes, degrees of freedom and *P* value noted<br>*Give P values as exact values whenever suitable.*
☒	☐	For Bayesian analysis, information on the choice of priors and Markov chain Monte Carlo settings
☒	☐	For hierarchical and complex designs, identification of the appropriate level for tests and full reporting of outcomes
☒	☐	Estimates of effect sizes (e.g. Cohen's *d*, Pearson's *r*), indicating how they were calculated

Our web collection on statistics for biologists contains articles on many of the points above.

Software and code

Policy information about availability of computer code

Data collection	FACS: BD FACSDiva 9; Immunofluorescence: Harmony 4.9; Illumina: NextSeq and NovaSeq OS; RT-qPCR: LightCycler 480 1.5.1; PromethION OS
Data analysis	Data analysis was performed with custom code made with Snakemake. All the necessary scripts and steps are found on a GitHub repository (https://github.com/NinoPandiloski/DNA-methylation-governs-the-sensitivity-of-repeats-to-restriction-by-the-HUSH-MORC2-corepressor). Versions of tools used in the analysis: STAR aligner (v 2.6.0), SAMtools (v 1.9), bamCoverage (v 2.4.3), featureCounts (v 1.6.3), DESeq (v 1.38.3), Bowtie2 (v 2.3.4.2), HOMER findPeaks (v 4.10), HOMER makeTagDirectory (v 4.10), DeepTools (v 2.5.4), minimap2 (v 2.24), nanopolish (v 0.13.2), SEACR (v 1.3), methylartist (v 1.2.4), TLDR (https://github.com/adamewing/tldr).

For manuscripts utilizing custom algorithms or software that are central to the research but not yet described in published literature, software must be made available to editors and reviewers. We strongly encourage code deposition in a community repository (e.g. GitHub). See the Nature Portfolio guidelines for submitting code & software for further information.

Data

Policy information about availability of data

All manuscripts must include a data availability statement. This statement should provide the following information, where applicable:

- Accession codes, unique identifiers, or web links for publicly available datasets
- A description of any restrictions on data availability
- For clinical datasets or third party data, please ensure that the statement adheres to our policy

There are no restrictions on data availability. The RNA and DNA sequencing data presented in this study have been deposited at GEOs:

GSE242143 (https://www.ncbi.nlm.nih.gov/geo/query/acc.cgi?acc=GSE242143) bulk RNA-seq, CUT&RUN and ONT whole genome sequencing of hNPC CRISPRi and control cells;
GSE224747 (https://www.ncbi.nlm.nih.gov/geo/query/acc.cgi?acc=GSE224747) 3' single nuclei RNAseq, CUT&RUN and bulk RNAseq of fetal tissue samples;
GSE209552 (https://www.ncbi.nlm.nih.gov/geo/query/acc.cgi?acc=GSE209552) 3' single nuclei RNAseq of adult post-mortem samples;
GSE211871 (https://www.ncbi.nlm.nih.gov/geo/query/acc.cgi?acc=GSE211871) Adult post-mortem NeuN+ CUT&RUN sequencing.
Source data are provided with this paper. Code has been deposited on GitHub (https://github.com/NinoPandiloski/DNA-methylation-governs-the-sensitivity-of-repeats-to-restriction-by-the-HUSH-MORC2-corepressor).

Research involving human participants, their data, or biological material

Policy information about studies with human participants or human data. See also policy information about sex, gender (identity/presentation), and sexual orientation and race, ethnicity and racism.

Reporting on sex and gender	Sex and gender were not considered in the study design. The CUT&RUN experiments on human material were restricted to qualitative analysis via genome browser tracks and summary heatmaps. No conclusions were based on quantitative analysis of these samples (n=2 for fetal and n=3 for adult post-mortem samples).
Reporting on race, ethnicity, or other socially relevant groupings	Race and ethnicity were not considered in the study design. The CUT&RUN experiments on human material were restricted to qualitative analysis via genome browser tracks and summary heatmaps. No conclusions were based on quantitative analysis of these samples (n=2 for fetal and n=3 for adult post-mortem samples).
Population characteristics	*Describe the covariate-relevant population characteristics of the human research participants (e.g. age, genotypic information, past and current diagnosis and treatment categories). If you filled out the behavioural & social sciences study design questions and have nothing to add here, write "See above."*
Recruitment	*Describe how participants were recruited. Outline any potential self-selection bias or other biases that may be present and how these are likely to impact results.*
Ethics oversight	The research performed on human tissue material was performed according to the highest ethical standards and in agreement with Swedish legislation. All experiments were approved by the Lund/Malmö Ethical Committee. Written consent was obtained and the participants were informed about the purpose of the research. No financial compensation was provided for tissue donors. Human fetal forebrain tissue was obtained from material available following elective termination of pregnancy at the University Hospital in Malmö, Sweden, according to guidelines approved by the Lund/Malmö Ethical Committee under the national ethical permit (reference number: Dnr 6.1.8-2887/2017). Human post-mortem brain tissue was obtained from the University Hospital in Lund, Sweden, according to guidelines approved by the Lund/Malmö Ethical Committee, under the national ethical permit (reference number: Dnr 2019-06582, 2020-02-12). The data were anonymised and the authors did not have access to identifiable information. Tissue data were processed on a sensitive offline computer cluster with special requirements for system access.

Note that full information on the approval of the study protocol must also be provided in the manuscript.

Field-specific reporting

Please select the one below that is the best fit for your research. If you are not sure, read the appropriate sections before making your selection.

[x] Life sciences   [ ] Behavioural & social sciences   [ ] Ecological, evolutionary & environmental sciences

For a reference copy of the document with all sections, see nature.com/documents/nr-reporting-summary-flat.pdf

Life sciences study design

All studies must disclose on these points even when the disclosure is negative.

Sample size	For transcriptomic analysis, sample size of at least n=3 (usually n=4) for both the control and CRISPRi groups was chosen. For comparative epigenomic analyses, experiments were typically repeated independently at least once, more for critical analyses (e.g. H3K9me3 quantitative differential occupancy analysis). The replicates are extensively described in the figure legends.
Data exclusions	One sample from the MORC2 CRISPRi transcriptome analysis was excluded. Prior to sequencing, this sample was noted to have lower RNA quality and a different cDNA profile by capillary electrophoresis. Following sequencing, the same sample deviated from others in the group based on PCA, and had significantly lower counts. In these situations the practice in the lab is to exclude the sample from the analysis.
Replication	CRISPRi experiments were repeated with at least two separate gRNAs, and the data pooled in differential analysis to limit the influence of off-target effects of gRNA-dCas9 recruitment. For analysis of HUSH-MORC2 corepressor targets, experiments from TASOR and MORC2 knockdowns were pooled. CRISPRi experiments with these factors have been replicated repeatedly by at least two individuals with different lentiviral batches. Lentiviral batches were validated by RT-qPCR and FACS. Transcriptomic data were orthogonally validated with short- and long-read RNA/cDNA-seq and H3K4me3 CUT&RUN analysis of promoters. All attempts at replication were successful provided efficient transduction of the cells was achieved.
Randomization	n/a

Blinding	Investigators were not blinded to the group allocation during data collection and analysis. Data analysis in this study was not deemed subjective.

Reporting for specific materials, systems and methods

We require information from authors about some types of materials, experimental systems and methods used in many studies. Here, indicate whether each material, system or method listed is relevant to your study. If you are not sure if a list item applies to your research, read the appropriate section before selecting a response.

Materials & experimental systems

n/a	Involved in the study
☐	☒ Antibodies
☐	☒ Eukaryotic cell lines
☒	☐ Palaeontology and archaeology
☒	☐ Animals and other organisms
☒	☐ Clinical data
☒	☐ Dual use research of concern
☒	☐ Plants

Methods

n/a	Involved in the study
☐	☒ ChIP-seq
☐	☒ Flow cytometry
☒	☐ MRI-based neuroimaging

Antibodies

Antibodies used	Rabbit anti-MORC2 (A300-149A, Bethyl; 1:1,000 dilution), rabbit anti-TASOR (Atlas HPA006735; 1:500), mouse anti-L1-ORF1p (Millipore MABC1152, 1:1,000) and HRP-conjugated anti--actin (Sigma A3854, 1:50,000) were used for Western blots. Rabbit anti-H3K4me3 (Active Motif 39159), rabbit anti-H3K9me3 (abcam 8898), rabbit anti-MORC2 (as above) and goat anti-rabbit IgG (abcam ab97047) were used for CUT&RUN assays (1:50-1:100 dilution). Rabbit anti-Nestin (Millipore AB5922, 1:100), goat anti-SOX2 (R&D Systems AF2018, 1:100), mouse anti-beta-III-tubulin (Biolegend 801202, 1:500), chicken anti-GFP (abcam 13970, 1:100), mouse anti-5mC (Active Motif 39649, 1:250), Alexa647 anti-rabbit (Jackson Immuno Research 711-605-152, 1:500), Alexa647 anti-mouse (Jackson Immuno Research 115-605-003, 1:500), donkey anti-goat Cy3 (Jackson ImmunoResearch 705-165-003, 1:200) and Alexa488 anti-chicken (Jackson Immuno Research, 703-546-155, 1:500) were used for immunocytochemistry.
Validation	All antibodies used in this study are routinely used in our lab:<br>- We have previously validated the Nestin and SOX2 antibodies in human embryonic tissue.<br>- The MORC2 Western blot (comparing control to CRISPRi KD) is itself a validation of the MORC2 antibody, which has also been validated elsewhere in ChIP and WB (using MORC2 KO clones as controls) as described at the manufacturer website.<br>- The 5mC staining was previously validated in our NPC cultures by comparing control and DNMT1-KO cells (where 5mC was absent - see Jönsson et al Nature Comms 2019).<br>- The H3K4me3, H3K9me3, L1 ORF1p, and TASOR antibodies are extensively validated in the literature and at the manufacturer's website.

Eukaryotic cell lines

Policy information about cell lines and Sex and Gender in Research

Cell line source(s)	The Sai2 human neuroepilethial-like stem cell line (male) was obtained from Anna Falk (Karolinska Institute, Stockholm, Sweden; current address Lund Stem Cell Center). Information here: https://www.cellosaurus.org/CVCL_A5DT and at PMID: 23884946
Authentication	We received the cells at low passage number directly from the Falk lab who derived these lines. The cells were characterized as human NPCs by RNAseq, ICC and by differentiation into neurons, as described in the paper and published literature.
Mycoplasma contamination	Cultures were tested routinely for Mycoplasma infection using Eurofins Mycoplasmacheck and returned a negative test.
Commonly misidentified lines (See ICLAC register)	No commonly misidentified cell lines were used.

Plants

Seed stocks	*Report on the source of all seed stocks or other plant material used. If applicable, state the seed stock centre and catalogue number. If plant specimens were collected from the field, describe the collection location, date and sampling procedures.*
Novel plant genotypes	*Describe the methods by which all novel plant genotypes were produced. This includes those generated by transgenic approaches, gene editing, chemical/radiation-based mutagenesis and hybridization. For transgenic lines, describe the transformation method, the number of independent lines analyzed and the generation upon which experiments were performed. For gene-edited lines, describe the editor used, the endogenous sequence targeted for editing, the targeting guide RNA sequence (if applicable) and how the editor was applied.*

Authentication

Describe any authentication procedures for each seed stock used or novel genotype generated. Describe any experiments used to assess the effect of a mutation and, where applicable, how potential secondary effects (e.g. second site T-DNA insertions, mosiacism, off-target gene editing) were examined.

ChIP-seq

Data deposition

- [x] Confirm that both raw and final processed data have been deposited in a public database such as GEO.
- [x] Confirm that you have deposited or provided access to graph files (e.g. BED files) for the called peaks.

Data access links
May remain private before publication.

https://www.ncbi.nlm.nih.gov/geo/query/acc.cgi?acc=GSE242143

Files in database submission

CUT&RUN files:
GSM7697250 CR117_LV3599_Control_IgG_rep1
GSM7697251 CR117_LV3599_Control_IgG_rep2
GSM7697252 CR112_LV3599_Control_IgG_rep3
GSM7697253 CR112_LV3599_Control_H3K9me3_rep1
GSM7697254 CR112_LV3599_Control_H3K9me3_rep2
GSM7697255 CR117_LV3599_Control_H3K9me3_rep3
GSM7697256 CR117_LV3599_Control_H3K4me3_rep1
GSM7697257 CR117_LV3599_Control_H3K4me3_rep2
GSM7697258 CR112_LV3688_MORC2_CRISPRi_IgG_rep1
GSM7697259 CR117_LV3691_TASOR_CRISPRi_IgG_rep1
GSM7697260 CR112_LV3688_MORC2_CRISPRi_H3K9me3_rep1
GSM7697261 CR112_LV3688_MORC2_CRISPRi_H3K9me3_rep2
GSM7697262 CR117_LV3688_MORC2_CRISPRi_H3K4me3_rep1
GSM7697263 CR117_LV3688_MORC2_CRISPRi_H3K4me3_rep2
GSM7697264 CR117_LV3691_TASOR_CRISPRi_H3K9me3_rep1
GSM7697265 CR117_LV3691_TASOR_CRISPRi_H3K9me3_rep2
GSM7697266 CR117_LV3691_TASOR_CRISPRi_H3K4me3_rep1
GSM7697267 CR117_LV3691_TASOR_CRISPRi_H3K4me3_rep2
GSM7697268 CR114_LV3393_Control_IgG_rep1
GSM7697269 CR114_LV3393_Control_IgG_rep2
GSM7697270 CR114_LV3393_Control_H3K9me3_rep1
GSM7697271 CR114_LV3393_Control_H3K9me3_rep2
GSM7697272 CR114_LV3393_Control_H3K4me3_rep1
GSM7697273 CR114_LV3393_Control_H3K4me3_rep2
GSM7697274 CR114_LV3367_DNMT1_CRISPRcut_IgG_rep1
GSM7697275 CR114_LV3367_DNMT1_CRISPRcut_IgG_rep2
GSM7697276 CR114_LV3367_DNMT1_CRISPRcut_H3K9me3_rep1
GSM7697277 CR114_LV3367_DNMT1_CRISPRcut_H3K9me3_rep2
GSM7697278 CR114_LV3367_DNMT1_CRISPRcut_H3K4me3_rep1
GSM7697279 CR114_LV3367_DNMT1_CRISPRcut_H3K4me3_rep2
GSM7697280 CR136_Sai2_untransduced_midXL_IgG
GSM7697281 CR136_Sai2_untransduced_midXL_MORC2
GSM7697282 CR137_LV3599_Control_midXL_IgG
GSM7697283 CR137_LV3599_Control_midXL_MORC2
GSM7697284 CR137_LV3800_DNMT1_CRISPRi_midXL_IgG
GSM7697285 CR137_LV3800_DNMT1_CRISPRi_midXL_MORC2
GSM8170402 CR153_LV3599_Control_H3K4me3_rep1
GSM8170403 CR153_LV3599_Control_H3K4me3_rep2
GSM8170404 CR153_LV3800_DNMT1_CRISPRi_H3K4me3_rep1
GSM8170405 CR153_LV3800_DNMT1_CRISPRi_H3K4me3_rep2
GSM8170406 LV3833-LV3688_DNMT1_MORC2_CRISPRi_H3K4me3_rep1
GSM8170407 LV3833-LV3688_DNMT1_MORC2_CRISPRi_H3K4me3_rep2

Genome browser session
(e.g. UCSC)

n/a

Methodology

Replicates

We performed CUT&RUN profiling of H3K9me3 and H3K4me3 and on MORC2 chromatin binding in various CRISPR-modified cell lines specified in the paper. In NPCs we produced at least 2 replicates for each of the epigenetic marks and for MORC2 binding. In fetal tissue and adult cortical (NeuN+) neurons we performed epigenome profiling from 2-3 individuals. In all cases we limited ourselves to qualitative analysis (e.g. comparing NPCs to the fetal tissue), with the exception of differential H3K9me3 occupancy analysis in Control (n=4), MORC2 (n=2) and TASOR (n=2) CRISPRi hNPCs where we had better power. In the latter analysis MORC2 and TASOR CRISPRi treatments were pooled to make n=4 in the treatment group. Note that quantitative analysis of separate MORC2 and TASOR

CRISPRi treatments was included in the Supplementary Information only upon reviewers requesting this, and showed similar effects. All experiments had a respective non-targeting IgG control. Experiments profiling H3K4me3 and H3K9me3 in control and DNMT1 CRISPRi or CRISPR-cut groups were repeated at least once with similar results.

Sequencing depth

Paired end approaches were used in all experiments.
Sample Total number of reads Uniquely mapped reads
GSM7697250 CR117_LV3599_Control_IgG_rep1 33310001 30383685
GSM7697251 CR117_LV3599_Control_IgG_rep2 24981584 22792031
GSM7697252 CR112_LV3599_Control_IgG_rep3 16815568 15579404
GSM7697253 CR112_LV3599_Control_H3K9me3_rep1 25202910 20501194
GSM7697254 CR112_LV3599_Control_H3K9me3_rep2 20900898 17121616
GSM7697255 CR117_LV3599_Control_H3K9me3_rep3 25607728 21706904
GSM7697256 CR117_LV3599_Control_H3K4me3_rep1 23017787 21933353
GSM7697257 CR117_LV3599_Control_H3K4me3_rep2 27372013 26005058
GSM7697258 CR112_LV3688_MORC2_CRISPRi_IgG_rep1 18986186 17710504
GSM7697259 CR117_LV3691_TASOR_CRISPRi_IgG_rep1 21786239 19958079
GSM7697260 CR112_LV3688_MORC2_CRISPRi_H3K9me3_rep1 17319822 14363982
GSM7697261 CR112_LV3688_MORC2_CRISPRi_H3K9me3_rep2 18484942 15307932
GSM7697262 CR117_LV3688_MORC2_CRISPRi_H3K4me3_rep1 38338961 36223137
GSM7697263 CR117_LV3688_MORC2_CRISPRi_H3K4me3_rep2 54272944 51645292
GSM7697264 CR117_LV3691_TASOR_CRISPRi_H3K9me3_rep1 24632025 20616983
GSM7697265 CR117_LV3691_TASOR_CRISPRi_H3K9me3_rep2 29071773 24311272
GSM7697266 CR117_LV3691_TASOR_CRISPRi_H3K4me3_rep1 17428340 16612224
GSM7697267 CR117_LV3691_TASOR_CRISPRi_H3K4me3_rep2 29092239 27564356
GSM7697268 CR114_LV3393_Control_IgG_rep1 24550246 22483180
GSM7697269 CR114_LV3393_Control_IgG_rep2 27362305 25096111
GSM7697270 CR114_LV3393_Control_H3K9me3_rep1 25330976 20867854
GSM7697271 CR114_LV3393_Control_H3K9me3_rep2 24091118 19718065
GSM7697272 CR114_LV3393_Control_H3K4me3_rep1 28483280 27214680
GSM7697273 CR114_LV3393_Control_H3K4me3_rep2 20455429 19516581
GSM7697274 CR114_LV3367_DNMT1_CRISPRcut_IgG_rep1 27172800 25151374
GSM7697275 CR114_LV3367_DNMT1_CRISPRcut_IgG_rep2 32712143 30221741
GSM7697276 CR114_LV3367_DNMT1_CRISPRcut_H3K9me3_rep1 23545673 20904739
GSM7697277 CR114_LV3367_DNMT1_CRISPRcut_H3K9me3_rep2 24055550 21457758
GSM7697278 CR114_LV3367_DNMT1_CRISPRcut_H3K4me3_rep1 18193452 17383072
GSM7697279 CR114_LV3367_DNMT1_CRISPRcut_H3K4me3_rep2 21133052 20183633
GSM7697280 CR136_Sai2_untransduced_midXL_IgG 9474630 8600518
GSM7697281 CR136_Sai2_untransduced_midXL_MORC2 20514164 18984206
GSM7697282 CR137_LV3599_Control_midXL_IgG 7049316 6438424
GSM7697283 CR137_LV3599_Control_midXL_MORC2 31519708 29822174
GSM7697284 CR137_LV3800_DNMT1_CRISPRi_midXL_IgG 18693856 17800552
GSM7697285 CR137_LV3800_DNMT1_CRISPRi_midXL_MORC2 9615690 8780164
GSM8170402 CR153_LV3599_Control_H3K4me3_rep1
GSM8170403 CR153_LV3599_Control_H3K4me3_rep2
GSM8170404 CR153_LV3800_DNMT1_CRISPRi_H3K4me3_rep1
GSM8170405 CR153_LV3800_DNMT1_CRISPRi_H3K4me3_rep2
GSM8170406 LV3833-LV3688_DNMT1_MORC2_CRISPRi_H3K4me3_rep1
GSM8170407 LV3833-LV3688_DNMT1_MORC2_CRISPRi_H3K4me3_rep2

Antibodies

Rabbit anti-MORC2 (A300-149A, Bethyl)
Rabbit anti-H3K4me3 (Active Motif 39159)
Rabbit anti-H3K9me3 (abcam 8898)
Goat anti-Rabbit IgG (abcam ab97047)

Peak calling parameters

Mapping was performed with Bowtie2 using the following mapping parameters:
[--local --very-sensitive-local --no-unal --no-mixed --no-discordant --phred33 -I 10 -X 700]
Peak Calling was performed with HOMER with the following parameters:
[findPeaks <control_H3K9me3_signal_tagDir> -style histone -o <output_baseline_peaks.txt> -i <control_IgG_signal_tagDir>]

Data quality

Pre- and post-library DNA was analysed by capillary electrophoresis and, for histone marks, showed nucleosomal patterns characteristic of successful CUT&RUN experiments. After sequencing FastQC was performed for each sample to ensure read quality. Mapping statistics from Bowtie2 were analysed to confirm that libraries did not contain an excessive fraction of PCR duplicates. To strike a balance between unique mapping and data quality, we set a MAPQ threshold of 10 for retaining alignments in instances where downstream analysis compared individual loci (e.g. in heatmaps, peak calling or genome browser snapshots). Peaks were called with HOMER with the standard settings [pVal > 0.0001 and FoldChange > 3]. Datasets were carefully inspected in a genome browser to ensure high and consistent quality across experimental groups, peaks over "positive control" regions (e.g. H3K4me3 over housekeeping genes; H3K9me3 over ZNF 3' exons) and where a CRISPRi treatment was made one could validate this internally (H3K9me3 over the promoter of the targeted gene; loss of H3K4me3 over the promoter of the targeted gene).

Software

The code for analysis was deposited on the following GitHub repository (https://github.com/NinoPandiloski/DNA-methylation-

governs-the-sensitivity-of-repeats-to-restriction-by-the-HUSH-MORC2-corepressor).

Flow Cytometry

Plots

Confirm that:

- [ ] The axis labels state the marker and fluorochrome used (e.g. CD4-FITC).
- [ ] The axis scales are clearly visible. Include numbers along axes only for bottom left plot of group (a 'group' is an analysis of identical markers).
- [ ] All plots are contour plots with outliers or pseudocolor plots.
- [ ] A numerical value for number of cells or percentage (with statistics) is provided.

Methodology

Sample preparation	The cells were de-attached, resuspended in media containing PI for dead cell exclusion and filtered with a 70um filter.
Instrument	BD Biosciences FACS Aria III
Software	Diva 9.0
Cell population abundance	A minimum of 200 cells were sorted into a tube containing PBS and PI. The sorted cells were reanalyzed and the % GFP positive cells out of live cells was determined and always at least 98%
Gating strategy	Live cells were gated for using PI. The GFP positive gate was set based on untransduced cells vs GFP expressing cells. The GFP + cells were validated to be the correct population by both ICC and sequencing.

- [x] Tick this box to confirm that a figure exemplifying the gating strategy is provided in the Supplementary Information.
